# The diverse echinostomes from East Africa: With a focus on species that use *Biomphalaria* and *Bulinus* as intermediate hosts

**DOI:** 10.1016/j.actatropica.2019.01.025

**Published:** 2019-05

**Authors:** Martina R. Laidemitt, Sara V. Brant, Martin W. Mutuku, Gerald M. Mkoji, Eric S. Loker

**Affiliations:** a Department of Biology, Center for Evolutionary and Theoretical Immunology, Museum of Southwestern Biology, University of New Mexico, 1 University of New Mexico, MSC03 2020, Albuquerque, NM, 87131, USA; b Center for Biotechnology Research and Development, Kenya Medical Research Institute (KEMRI), P.O. Box 54840-00200, Nairobi, Kenya

**Keywords:** Biodiversity, Echinostomatidae, DNA barcode, Echinostomiasis, Schistosoma

## Abstract

Echinostomes are a diverse group of digenetic trematodes that are globally distributed. The diversity of echinostomes in Africa remains largely unknown, particularly in analyses using molecular markers. Therefore, we were interested in the composition and host usage patterns of African echinostomes, especially those that also use schistosome transmitting snails as intermediate hosts. We collected adults and larval stages of echinostomes from 19 different localities in East Africa (1 locality in Uganda and 18 in Kenya). In this study we provide locality information, host use, museum vouchers, and genetic data for two loci (28S and *nad*1) from 98 samples of echinostomes from East Africa. Combining morphological features, host use information, and phylogenetic analyses we found 17 clades of echinostomes in East Africa. Four clades were found to use more than one genus of freshwater snails as their first intermediate hosts. We also determined at least partial life cycles (2 of the 3) of four clades using molecular markers. Of the 17 clades, 13 use *Biomphalaria* or *Bulinus* as a first intermediate host. The overlap in host usage creates opportunities for competition, including against human schistosomes. Thus, our study can be used as a foundation for future studies to ascertain the interactions between schistosomes and echinostomes in their respective intermediate hosts.

## Highlights

• 17 different clades of echinostomes were found in East Africa.
• The majority (13/17) of clades use *Biomphalaria* or *Bulinus* as an intermediate host.
• For four clades partial life cycles (2/3) were determined.

## 1 Introduction

The Echinostomatoidea is a diverse superfamily of trematodes that includes nine families and 105 genera (Tkach et al., 2016). Here we focus on taxa from one of the families the Echinostomatidae, referred to hereafter as echinostomes. Echinostomes are globally distributed and have a multi-host life cycle that involves a vertebrate definitive host, a molluscan first intermediate host, and a second intermediate host that is typically a mollusc, amphibian, or fish. Echinostomes are known to cause disease in humans, mostly in southeast Asia where raw second intermediate hosts are consumed (Graczyk and Fried, 1998). Echinostomes are also known to actively influence the establishment of pre-existing infections in snail first intermediate hosts, thus are considered important components to community composition over time and space (Lim and Heyneman, 1972; Sousa, 1993; Lafferty et al., 1994; Sapp et al., 1998; Hechinger et al., 2011).

Echinostomes are characterized by having a distinctive cephalic crown of collar spines, a ventral sucker larger than the oral sucker, two testes tandemly or symmetrically arranged, a pretesticular ovary, and a cirrus sac (Fried, 2001; Kostadinova and Jones, 2005; Fried and Toledo, 2009). The family Echinostomatidae (the recent reclassification now includes taxa that belonged to the former Rhopaliidae, Looss, 1899; Cathaemasiidae Fuhrmann, 1928; and Ribeiroiinae Travassos, 1951) is the most speciose family in the superfamily (Tkach et al., 2016). Delineation of genera has traditionally been based extensively on characteristics of adult worms and has included consideration of definitive host use, the morphology of the cephalic collar, number and arrangement of the collar spines, position of the testes and ovary, and location and structure of the vitellaria (Kostadinova, 2005). Characteristics of the larval stages, especially of cercariae, have received less consideration. A recent molecular phylogenetic study focused on 28S rRNA sequences and incorporated a broad array of echinostome species has provided a new framework to organize our thinking about echinostomes (Tkach et al., 2016); however, while most continents have some representative taxa, echinostomes from Africa are largely lacking.

The focus of this study is to uncover the diversity of echinostomes transmitted in Africa as part of a larger focus in how biodiversity can influence disease transmission. One of our motivations is to learn how other digenean species, particularly echinostomes, can influence schistosomiasis transmission in Sub-Saharan Africa by competing with schistosome sporocysts for access to their required snail hosts. Studies have shown that when multiple species of digenetic trematodes colonize the same snail host, echinostomes have usually proven to be dominant to other species (Lim and Heyneman, 1972; Hechinger et al., 2011). There is growing evidence for some species that echinostome rediae are specialized for the purpose of attacking and killing the larvae of competing digenean species within the body of their molluscan hosts, while other rediae are more specialized for reproduction (Garcia-Vedrenne et al., 2016). An important first step in understanding echinostome-schistosome interactions is the characterization of the biodiversity and host use of echinostome species in East Africa. With these data, we can relate our results to the growing body of research that highlights the relevance between biodiversity and human disease transmission (Johnson and Thieltges, 2010; Civitello et al., 2015).

However, very little is known about African echinostomes with respect to biogeography, phylogenetic placement (especially using molecular markers), and host use. The majority of echinostome descriptions from Africa are of adults that use birds as a definitive host (Dietz, 1909; Odhner, 1910; Faust, 1921; Hilmy, 1949; Dollfus, 1950; Bisseru, 1957; Appleton et al., 1983; King and As, 2000). Even meticulous species descriptions of adult morphology alone can lead to confusion in their systematics. Likewise, descriptions other life cycle stages like cercariae or metacercariae by themselves may also prove difficult or unreliable as a basis for species identifications. Therefore, to characterize the species diversity of echinostomes in Kenya and surrounding areas, particularly in the Lake Victoria Basin which encompasses multiple East African countries, molecular markers were used in combination with morphological features to identify the life cycle stages we collected. This effort allowed us to link certain life cycle stages across space and time to better differentiate clades of echinostomes and understand host usage patterns and how they relate to disease transmission in East Africa.

Towards this end, we collected and characterized different species of echinostomes that are transmitted in East Africa, primarily from western Kenya, with an emphasis on species that use *Biomphalaria* or *Bulinus* as their first intermediate hosts. These two snail genera host *Schistosoma mansoni,* and *S. haematobium* and its close relatives, respectively. Our goal here is to detail how many echinostome species use these snail hosts to provide context for future experiments to determine their ability to compete with and prey upon the sporocysts of schistosomes in their snail hosts. It is also critical for the evolutionary characterization of organisms to have a permanent museum voucher (Pleijel et al., 2008; Valkiunas et al., 2008; Hoberg et al., 2009), thus we provide vouchered materials that include locality information, sequence data, provisional species identification, and host use information for the African echinostome adults and larvae collected.

## 2 Materials and methods

### 2.1 Sampling

All field-collected aquatic snails were brought to the lab and were placed individually into 12-well tissue culture plates in 3 ml of aged tap water. The tissue culture plates were placed in natural light for two hours to induce shedding of cercariae. Available keys were used for preliminary identification of African snails and their trematodes (Fain, 1953; Brown 1984; Brown and Kristensen, 1989; Brown, 1994; Frandsen and Christensen, 1984; Schell, 1985). Cercariae and rediae were fixed in 95% ethanol for later molecular analysis.

### 2.2 Staining adult worms

Adult worms were preserved in 95% ethanol and then were placed into 70% ethanol for 24 h prior to staining. To maintain a connection between the morphological voucher and the genetic voucher, part of the posterior portion of the adult was severed and used for molecular work and the remaining part was stained according to Fried and Manger (1992).

### 2.3 Molecular characterization

We sequenced the 28S gene because such data are available for many of the echinostomes listed in GenBank and can thus provide a broader taxonomic comparison of our specimens into genera according to the scheme of Tkach et al. (2016). We sequenced the *nad*1 gene to provide additional resolution for some of the more-closely related representatives we obtained. Partial sequences of the 28S ribosomal gene and nicotinamide adenine dinucleotide dehydrogenase subunit 1 (*nad*1) from 98 echinostome specimens were amplified by polymerase chain reaction (PCR). Samples were chosen based on host usage, locality, and sampling year. One or two cercariae, one rediae, or a partial portion of the posterior end of an adult were used for DNA extraction. Genomic DNA was extracted using the QIAamp DNA Micro Kit following the manufacturer’s instructions, with a final elution volume of 35 μl (Qiagen, Valencia, CA).

The 28S gene was amplified using forward primer, dig12 (5′-AAG CAT ATC ACT AAGCGG-3′) and reverse primer 1500R (5′-GCT ATC CTG AGGGAA ACT TCG-3′) (Tkach et al., 2003). The volume of each PCR reaction was 25 μl with 1 μl of 100 ng of DNA, 0.8 mM/l dNTPs, 2.5 mM/l MgCl2, 0.25 units of Ex Taq DNA (Clontech, Mountain View, CA), and 0.4 μM/L of each primer. PCR cycles were followed according to Tkach et al. (2016).

The *nad*1 gene was amplified using forward primer NDJ11 (Morgan and Blair, 1998) (5′ -AGA TTCGTA AGG GGC CTA ATA-3′) and the reverse primer NDJ2a (5′-CTT CAG CCT CAG CAT AAT-3′) (Kostadinova et al., 2003). The volume of each PCR reaction was 25 μl with 1 μl of 100 ng of DNA, 0.8 mM/l dNTPs, 2.5 mM/l MgCl2, 0.25 units of Ex Taq DNA (Clontech, Mountain View, CA), and 0.4 μM/L of each primer PCR cycles were performed on Eppendorf Mastercycler epigradient machines which were programmed as follows: 2 min denaturation at 94 °C; 94 °C for 1 min, 54 °C for 30 s and 72 °C for 1 min for three cycles; 94 °C for 1 min, 53 °C for 30 s, and 72 °C for 1 min for three cycles; 94 °C for 1 min, 52 °C for 30 s and 72 °C for 1 min for three cycles; 94 °C for 1 min, 51 °C for 30 s and 72 °C for 1 min for 20 cycles, and followed by an extension step for 7 min at 72 °C.

For some of the samples, only the cercariae (after shedding of snails) were saved, but its snail host was not preserved. Cercariae can often have adherent snail tissue on them that can be amplified with snail specific primers (Devkota et al., 2015). Therefore, for the cercariae where we did not have the snail host, we used snail *cox1* primers to generate amplicons from those cercariae, particularly those shed from *Bulinus*. This was done in attempt to verify the *Bulinus* species from which the cercariae were shed, because identification based only on snail keys is difficult for this genus (Stothard et al., 2002). Many of samples yielded amplicons; however, in some cases, we were unable to amplify snail DNA from the cercariae samples, therefore we could not designate a species.

PCR fragments were separated by agarose gel electrophoresis and visualized with 0.5% GelRed^™^ Nucleic acid gel stain (Biotium, Hayward, CA, USA). PCR products were purified using the Illustra ExoProStar (GE Healthcare Life Sciences, Pittsburgh, PA). Both strands were sequenced using an Applied Biosystems 3130 automated sequencer and BigDye terminator cycle sequencing kit Version 3.1 (Applied Biosystems, Foster City, CA). DNA sequences were verified by aligning reads from the 5′ and 3′ directions using Sequencher 5.0 and manually corrected for ambiguous base calls (Gene Codes, Ann Arbor, Michigan).

### 2.4 Sequence alignment and phylogenetic analyses

28S and *nad*1 sequences were used in phylogenetic analyses using Maximum Likelihood (ML) and Bayesian interferences (BI). The analysis included 47 specimens from NCBI-GenBank for 28S and 41 for *nad*1. Non-redundant sequences were aligned by eye and edited in MEGA7 (Kumar et al., 2016). A total of 1143 bases were used for 28S alignment and 493 bases for *nad*1 alignments. Sequences generated in this study were submitted to GenBank (Table 2). ML and BI analyses were carried out using PAUP* 4.0 b10 (Wilgenbusch and Swofford, 2003) and MrBayes v 3.12 (Ronquist and Huelsenbeck, 2003) respectively. jModelTest 2.0 (Darriba et al., 2012) was used to find the best fit model of substitution for BI and ML for both genes. Heuristic searchers were utilized for ML analyses and 1000 bootstrap replicates were run for each dataset. For BI analyses the parameters were unlinked: In both datasets the trees were sampled every 100 cycles, and the first 25% of trees with pre-asymptotic likelihood scores were discarded as burn-in.

Uncorrected pairwise distance values (*p*-distance) were calculated in MEGA7 (Kumar et al., 2016). Data were summarized within and between groups (Table 3, Table 4). We followed other studies in using a *p*-distance value >5% in mtDNA markers to provisionally designate our specimens as distinct species (Vilas et al., 2005; Brant and Loker, 2009; Detwiler et al., 2010; Laidemitt et al., 2017).

## 3 Results

### 3.1 Samples

We collected echinostome adults and larva between 2002–2017 from 19 localities (Table 1). Cercariae or rediae were collected from 9 species of snail hosts and adults were collected from two species of birds. We sequenced 28S and *nad*1 from 92 different cercariae, 4 metacercariae, and 2 adult samples. Although we attempted to sequence *nad*1 from all 98 samples, 4 samples would not amplify using the *nad*1 primers. Our specimens were deposited as vouchers in the Museum of Southwestern Biology (MSB).

**Table 1:** Collections localities.

Locality	Lat	Long
Sirikwa dam	0.46713	35.35170
Monitor Lizard Pond	−0.71659	37.32537
Anyanga Beach	−0.05364	34.05149
Asao Stream	−0.31810	35.00690
Dunga Beach	−0.14532	34.736330
Kasabong Stream	−0.15190	34.33550
Powerhouse Beach	−0.09410	34.70760
Carwash Beach	−0.09587	34.74850
Kazinga Channel	−0.191928	29.89807
Kameta Dam	−0.109979	34.77456
Nawa Beach	−0.10194	34.71333
Forest Beach	−0.356594	34.68358
Kabuong beach	−0.336198	34.356155
Kotieno Beach	−0.35250	34.66733
Mwea Rice Field	−0.81800	37.62200
Kagwa Beach	−0.356594	34.68358
Kobala Beach	−0.34864	34.689057
Alara Beach	−0.350466	34.753866, 34.75387

**Table 2:** Provisional identification, sample name, host it was collected from, life cycle stage, collection locality, date, Museum of Southwestern Biology voucher number, and GenBank accession numbers of echinostome specimens used in this study.

Provisonal ID	Sample Name	Host	Stage	Locality	Date Collected	MSB Voucher Number	GenBank 28S	GenBank *nad*1
*Petasiger sp.* 5	PE1	*Bulinus sp.*	Cercariae	Monitor Lizard Pond	Jan-14	MSB:Para:26602	MK482414	MK534340
*Petasiger sp.* 5	PE2	*Bulinus sp.*	Cercariae	Monitor Lizard Pond	Jan-14	MSB:Para:26620	MK482425	MK534348
*Petasiger sp.* 5	PE3	*Bulinus sp.*	Cercariae	Monitor Lizard Pond	Jan-14	MSB:Para:26644	MK482436	MK534355
Petasiger sp. 6	PE4	*Bulinus sp.*	Cercariae	Monitor Lizard Pond	Jan-14	MSB:Para:26655	MK482447	No amplicon
*Petasiger sp.* 3	PE5	*Bulinus sp.*	Cercariae	Sirikwa Dam	Jan-14	MSB:Para:26666	MK482458	MK534375
*Petasiger sp*. 4	PE6	*Biomphalaria pfeifferi*	Cercariae	Monitor Lizard Pond	Jan-14	MSB:Para:26677	MK482469	MK534385
*Petasiger sp.* 5	PE7	*Bulinus truncatus trigonus*	Cercariae	Anyanga Beach	Jan-17	MSB:Para:26688	MK482480	MK534396
*Patagifer sp.* 1	PE8	*Biomphalaria sudanica*	Cercariae	Dunga Beach	Apr-17	MSB:Para:26601	MK482491	MK534407
*Patagifer sp.* 1	PE9	*Biomphalaria pfeifferi*	Cercariae	Asao Stream	Jul-15	MSB:Para:26626	MK482502	MK534418
Echinostomatidae sp. 1	PE10	*Ceratophallus natalensis*	Cercariae	Asao Stream, Kenya	Jun-15	MSB:Para:26603	MK482415	MK534342
Echinostomatidae sp. 2	PE11	*Biomphalaria pfeifferi*	Cercariae	Asao Stream, Kenya	Jun-15	MSB:Para:26604	MK482416	MK534339
*Patagifer sp.* 1	PE12	*Biomphalaria sudanica*	Cercariae	Powerhouse Beach	Jan-13	MSB:Para:26605	MK482417	MK534343
*Ribeiroia sp*.2	PE13	*Biomphalaria sudanica*	Cercariae	Powerhouse Beach	Jan-14	MSB:Para:26606	MK482418	No amplicon
Echinostomatidae sp. 1	PE14	*Ceratophallus natalensis*	Cercariae	Carwash Beach	Aug-12	MSB:Para:26607	MK482419	MK534344
*Patagifer sp.* 1	PE15	*Ceratophallus natalensis*	Cercariae	Carwash Beach	Aug-12	MSB:Para:26608	MK482420	MK534341
Patagifer sp. 2	PE16	*Biomphalaria sudanica*	Cercariae	Dunga Beach	Apr-17	MSB:Para:26616	MK482421	MK534345
*Patagifer sp.* 1	PE17	*Biomphalaria sudanica*	Cercariae	Kazing Channel	May-02	MSB:Para:26617	MK482422	MK534335
*Patagifer sp.* 2	PE18	*Biomphalaria sudanica*	Cercariae	Kazing Channel	May-02	MSB:Para:26618	MK482423	MK534346
*Ribeiroia sp.* 1	PE19	*Biomphalaria sudanica*	Cercariae	Powerhouse Beach	May-02	MSB:Para:26619	MK482424	MK534347
*Patagifer sp. 1*	PE20	*Biomphalaria sudanica*	Cercariae	Dunga Beach	May-17	MSB:Para:26621	MK482426	MK534349
*Patagifer sp.* 1	PE21	*Biomphalaria sudanica*	Cercariae	Powerhouse Beach	Dec-10	MSB:Para:26622	MK482427	MK534336
*Patagifer sp.* 1	PE22	*Bulinus ugandae*	Cercariae	Powerhouse Beach	Jan-17	MSB:Para:26630	MK482428	MK534338
*Patagifer sp.* 1	PE23	*Biomphalaria sudanica*	Cercariae	Dunga Beach	Apr-17	MSB:Para:26631	MK482429	MK534337
*Petasiger sp*. 4	PE24	*Biomphalaria pfeifferi*	Cercariae	Mwea Rice Field	Jan-13	MSB:Para:26632	MK482430	MK534350
Echinostomatidae sp. 2	PE25	*Biomphalaria pfeifferi*	Cercariae	Asao Stream	Feb-13	MSB:Para:26633	MK482431	MK534351
*Patagifer sp.* 1	PE26	*Biomphalaria sudanica*	Cercariae	Carwash Beach	Jan-12	MSB:Para:26634	MK482432	MK534352
Echinostomatidae sp. 1	PE27	*Ceratophallus natalensis*	Cercariae	Powerhouse Beach	Aug-12	MSB:Para:26635	MK482433	MK534353
*Patagifer sp.* 1	PE28	*Biomphalaria sudanica*	Cercariae	Powerhouse Beach	Aug-12	MSB:Para:26636	MK482434	MK534354
*Ribeiroia sp*.2	PE29	*Biomphalaria pfeifferi*	Cercariae	Asao Stream	Oct-13	MSB:Para:26643	MK482435	No amplicon
Isthmiophora sp.	PE30	*Radix natalensis*	Cercariae	Nyamo Saro	Jun-05	MSB:Para:26645	MK482437	MK534356
*Patagifer sp.* 1	PE31	*Biomphalaria pfeifferi*	Cercariae	Kasabong Stream	Oct-13	MSB:Para:26646	MK482438	MK534357
*Ribeiroia sp*.2	PE32	*Biomphalaria pfeifferi*	Cercariae	Mwea Rice Field	Oct-13	MSB:Para:26647	MK482439	No amplicon
*Patagifer sp.* 1	PE33	*Biomphalaria sudanica*	Cercariae	Powerhouse Beach	Jan-13	MSB:Para:26648	MK482440	MK534358
Echinostomatidae sp. 2	PE34	*Microcarbo africanus*	Adult	Kameta Dam	Jan-05	MSB:Para:26649	MK482441	MK534359
Echinostomatidae sp. 2	PE35	*Biomphalaria pfeifferi*	Cercariae	Asao Stream	Jan-14	MSB:Para:26650	MK482442	MK534360
*Petasiger sp.* 1	PE36	*Radix natalensis*	Cercariae	Monitor Lizard Pond	Jan-14	MSB:Para:26651	MK482443	MK534361
*Petasiger sp*. 4	PE37	*Biomphalaria pfeifferi*	Cercariae	Mwea Rice Field	Jan-13	MSB:Para:26652	MK482444	MK534362
Echinostomatidae sp. 2	PE38	*Phalacrocorax africanus*	Adult	Kameta Dam	Jan-05	MSB:Para:26653	MK482445	MK534363
*Petasiger sp.* 3	PE39	*Radix natalensis*	Cercariae	Monitor Lizard Pond	Jan-14	MSB:Para:26654	MK482446	MK534364
*Patagifer sp.* 1	PE40	*Bulinus ugandae*	Cercariae	Powerhouse Beach	Jan-17	MSB:Para:26656	MK482448	MK534365
Petasiger sp. 2	PE41	*Bulinus globosus*	Cercariae	Asao Stream	Jan-17	MSB:Para:26657	MK482449	MK534366
Petasiger sp. 2	PE42	*Bulinus globosus*	Cercariae	Asao Stream	Jan-17	MSB:Para:26658	MK482450	MK534367
Petasiger sp. 2	PE43	*Bulinus globosus*	Cercariae	Asao Stream	Jan-17	MSB:Para:26659	MK482451	MK534368
Petasiger sp. 2	PE44	*Bulinus globosus*	Cercariae	Asao Stream	Jan-17	MSB:Para:26660	MK482452	MK534369
Petasiger sp. 2	PE45	*Bulinus globosus*	Cercariae	Asao Stream	Jan-17	MSB:Para:26661	MK482453	MK534370
Petasiger sp. 2	PE46	*Bulinus globosus*	Cercariae	Asao Stream	Jan-17	MSB:Para:26662	MK482454	MK534371
*Petasiger sp.* 5	PE47	*Bulinus globosus*	Cercariae	Asao Stream	Jan-17	MSB:Para:26663	MK482455	MK534372
*Patagifer sp.* 2	PE48	*Biomphalaria pfeifferi*	Cercariae	Asao Stream	Jan-17	MSB:Para:26664	MK482456	MK534373
*Patagifer sp.* 2	PE49	*Biomphalaria pfeifferi*	Cercariae	Asao Stream	Jan-17	MSB:Para:26665	MK482457	MK534374
Petasiger sp. 2	PE50	*Bulinus globosus*	Cercariae	Asao Stream	Apr-16	MSB:Para:26667	MK482459	MK534376
*Patagifer sp.* 1	PE51	*Biomphalaria sudanica*	Cercariae	Powerhouse Beach	Jul-16	MSB:Para:26668	MK482460	MK534377
*Ribeiroia sp.* 3	PE52	*Biomphalaria sudanica*	Cercariae	Powerhouse Beach	Aug-16	MSB:Para:26669	MK482461	No amplicon
Patagifer sp. 2	PE53	*Biomphalaria sudanica*	Cercariae	Powerhouse Beach	Aug-16	MSB:Para:26670	MK482462	MK534378
*Petasiger sp*. 4	PE54	*Biomphalaria sudanica*	Cercariae	Powerhouse Beach	Jun-16	MSB:Para:26671	MK482463	MK534379
*Patagifer sp.* 2	PE55	*Biomphalaria sudanica*	Cercariae	Powerhouse Beach	Jun-16	MSB:Para:26672	MK482464	MK534380
*Patagifer sp.* 2	PE56	*Biomphalaria pfeifferi*	Cercariae	Asao Stream	Jun-16	MSB:Para:26673	MK482465	MK534381
Patagifer sp. 1	PE57	*Biomphalaria sudanica*	Cercariae	Dunga Beach	Jun-16	MSB:Para:26674	MK482466	MK534382
*Petasiger sp*. 4	PE58	*Biomphalaria sudanica*	Cercariae	Powerhouse Beach	Jun-16	MSB:Para:26675	MK482467	MK534383
*Patagifer sp.* 1	PE59	*Bulinus ugandae*	Cercariae	Powerhouse Beach	Jan-15	MSB:Para:26676	MK482468	MK534384
Petasiger sp. 2	PE60	*Pila ovata*	Cercariae	Dunga Beach	Sep-15	MSB:Para:26678	MK482470	MK534386
Patagifer sp. 2	PE61	*Biomphalaria pfeifferi*	Metacercariae	Asao Stream	Jun-16	MSB:Para:26679	MK482471	MK534387
Patagifer sp. 2	PE62	*Biomphalaria pfeifferi*	Cercariae	Asao Stream	Jun-16	MSB:Para:26680	MK482472	MK534388
Echinostomatidae sp. 2	PE63	*Biomphalaria pfeifferi*	Cercariae	Asao Stream	Jun-16	MSB:Para:26681	MK482473	MK534389
*Patagifer sp.* 1	PE64	*Biomphalaria sudanica*	Cercariae	Powerhouse Beach	Jun-16	MSB:Para:26682	MK482474	MK534390
*Patagifer sp.* 1	PE65	*Bulinus ugandae*	Cercariae	Powerhouse Beach	Jun-16	MSB:Para:26683	MK482475	MK534391
*Patagifer sp.* 1	PE66	*Biomphalaria pfeifferi*	Cercariae	Kasabong Stream	Jan-15	MSB:Para:26684	MK482476	MK534392
Echinostomatidae sp. 2	PE67	*Biomphalaria pfeifferi*	Cercariae	Asao Stream	Aug-16	MSB:Para:26685	MK482477	MK534393
*Echinoparphium sp.*	PE68	*Bulinus tropicus*	Cercariae	Mwea Rice Field	Jan-15	MSB:Para:26686	MK482478	MK534394
*Patagifer sp. 1*	PE69	*Biomphalaria sudanica*	Cercariae	Ovara Beach	Apr-16	MSB:Para:26687	MK482479	MK534395
*Patagifer sp. 1*	PE70	*Biomphalaria sudanica*	Cercariae	Kagaw Beach	Apr-16	MSB:Para:26689	MK482481	MK534397
Echinostomatidae sp. 2	PE71	*Biomphalaria pfeifferi*	Cercariae	Asao Stream	Aug-16	MSB:Para:26690	MK482482	MK534398
Echinostomatidae sp. 2	PE72	*Biomphalaria pfeifferi*	Cercariae	Asao Stream	Aug-16	MSB:Para:26691	MK482483	MK534399
Echinostomatidae sp. 3	PE73	*Ceratophallus natalensis*	Cercariae	Asao Stream	Aug-16	MSB:Para:26594	MK482484	MK534400
*Patagifer sp. 1*	PE74	*Biomphalaria sudanica*	Cercariae	Powerhouse Beach	Jun-16	MSB:Para:26595	MK482485	MK534401
*Patagifer sp. 2*	PE75	*Biomphalaria sudanica*	Cercariae	Powerhouse Beach	Jun-16	MSB:Para:26596	MK482486	MK534402
*Patagifer sp.* 2	PE76	*Biomphalaria pfeifferi*	Cercariae	Asao Stream	Jun-16	MSB:Para:26597	MK482487	MK534403
Echinostomatidae sp. 2	PE77	*Biomphalaria pfeifferi*	Cercariae	Asao Stream	Jul-15	MSB:Para:26598	MK482488	MK534404
*Petasiger sp*. 4	PE78	*Biomphalaria sudanica*	Cercariae	Powerhouse Beach	Jan-16	MSB:Para:26599	MK482489	MK534405
*Echinostoma caproni*	PE79	*Biomphalaria sudanica*	Cercariae	Kabuong Beach	Jan-17	MSB:Para:26600	MK482490	MK534406
Echinostomatidae sp. 1	PE80	*Segmentorbis kanisaensis*	Cercariae	Nawa Beach	Jun-16	MSB:Para:26609	MK482492	MK534408
*Patagifer sp. 1*	PE81	*Bulinus ugandae*	Cercariae	Powerhouse Beach	Jan-17	MSB:Para:26610	MK482493	MK534409
*Petasiger sp*. 4	PE82	*Biomphalaria sudanica*	Cercariae	Kobala Beach	Sep-16	MSB:Para:26611	MK482494	MK534410
*Petasiger sp.* 5	PE83	*Bulinus ugandae*	Cercariae	Powerhouse Beach	Jan-16	MSB:Para:26612	MK482495	MK534411
*Patagifer sp. 1*	PE84	*Biomphalaria sudanica*	Cercariae	Powerhouse Beach	Jan-16	MSB:Para:26613	MK482496	MK534412
*Patagifer sp.* 2	PE85	*Biomphalaria pfeifferi*	Cercariae	Kasabong	Jan-16	MSB:Para:26614	MK482497	MK534413
*Patagifer sp. 1*	PE86	*Biomphalaria sudanica*	Cercariae	Powerhouse Beach	Jan-16	MSB:Para:26615	MK482498	MK534414
*Patagifer sp. 1*	PE87	*Biomphalaria sudanica*	Cercariae	Nawa Beach	Feb-17	MSB:Para:26623	MK482499	MK534415
*Petasiger sp*. 4	PE88	*Biomphalaria sudanica*	Cercariae	Dunga Beach	Feb-17	MSB:Para:26624	MK482500	MK534416
*Echinostoma caproni*	PE89	*Biomphalaria sudanica*	Cercariae	Kabuong Beach	Jan-17	MSB:Para:26625	MK482501	MK534417
*Patagifer sp. 1*	PE90	*Biomphalaria pfeifferi*	Cercariae	Asao Stream	Jul-15	MSB:Para:26627	MK482503	MK534419
*Patagifer sp. 1*	PE91	*Biomphalaria sudanica*	Cercariae	Forest Beach	Jan-17	MSB:Para:26628	MK482504	MK534420
*Patagifer sp. 1*	PE92	*Biomphalaria sudanica*	Metacercariae	Dunga Beach	Feb-17	MSB:Para:26629	MK482505	MK534421
*Patagifer sp. 1*	PE93	*Biomphalaria sudanica*	Metacercariae	Dunga Beach	Feb-17	MSB:Para:26642	MK482506	MK534422
*Patagifer sp. 1*	PE94	*Biomphalaria sudanica*	Metacercariae	Dunga Beach	Feb-17	MSB:Para:26637	MK482507	MK534423
*Ribeiroia sp*.2	PE95	*Biomphalaria sudanica*	Metacercariae	Dunga Beach	Feb-17	MSB:Para:26638	MK482508	No amplicon
*Patagifer sp. 1*	PE96	*Biomphalaria sudanica*	Cercariae	Kotieno Beach	Jan-17	MSB:Para:26639	MK482509	MK534424
Echinostomatidae sp. 2	PE97	*Biomphalaria pfeifferi*	Cercariae	Asao Stream	Jul-15	MSB:Para:26640	MK482510	MK534425
*Patagifer sp. 2*	PE98	*Biomphalaria pfeifferi*	Cercariae	Asao Stream	Jul-15	MSB:Para:26641	MK482511	MK534426

**Table 3:** Intra- and interclade P- distance values of 28S amplified from the 98 echinostomes in this study.

Clade Number	1	2	3	4	5	6	7	8	9	10	11	12	13	14	15	16	17
1	**0.001**																
2	0.020	**0.007**															
3	0.018	0.004	**0.001**														
4	0.020	0.024	0.022	**0.000**													
5	0.024	0.029	0.027	0.007	**0.004**												
6	0.024	0.028	0.026	0.009	0.013	**n/c**											
7	0.033	0.032	0.031	0.034	0.037	0.038	**n/c**										
8	0.061	0.057	0.059	0.062	0.067	0.064	0.066	**n/c**									
9	0.060	0.056	0.058	0.063	0.068	0.064	0.066	0.003	**0.000**								
10	0.063	0.060	0.061	0.066	0.071	0.068	0.068	0.012	0.008	**n/c**							
11	0.048	0.050	0.051	0.053	0.059	0.057	0.055	0.048	0.050	0.055	**n/c**						
12	0.052	0.055	0.056	0.056	0.062	0.060	0.058	0.054	0.055	0.059	0.020	**n/c**					
13	0.051	0.055	0.056	0.057	0.064	0.063	0.058	0.055	0.056	0.061	0.015	0.024	**0.001**				
14	0.049	0.052	0.052	0.053	0.060	0.059	0.055	0.054	0.056	0.056	0.018	0.024	0.020	**0.000**			
15	0.045	0.048	0.049	0.048	0.055	0.055	0.053	0.051	0.053	0.056	0.013	0.018	0.014	0.007	**0.002**		
16	0.049	0.050	0.051	0.054	0.061	0.059	0.056	0.052	0.053	0.055	0.022	0.027	0.019	0.020	0.015	**0.001**	
17	0.055	0.056	0.056	0.060	0.066	0.065	0.063	0.060	0.061	0.063	0.027	0.036	0.027	0.026	0.023	0.028	**n/c**

Bolded values are intraclade P-distance numbers.

**Table 4:** Intra- and interclade P- distance values of *nad1* amplified from the 94 (minus the 4 Ribeiroia samples) echinostomes in this study.

Clade Number	1	2	3	4	5	6	7	8	9	10	11	12	13	14
1	**0.002**													
2	0.184	**0.007**												
3	0.200	0.077	**0.015**											
4	0.171	0.166	0.184	**0.001**										
5	0.204	0.177	0.191	0.146	**0.010**									
6	0.177	0.150	0.160	0.149	0.165	**n/c**								
7	0.214	0.224	0.230	0.223	0.229	0.204	**n/c**							
8	0.302	0.307	0.305	0.320	0.309	0.291	0.332	**n/c**						
9	0.295	0.267	0.276	0.282	0.285	0.279	0.291	0.334	**n/c**					
10	0.284	0.274	0.265	0.279	0.271	0.275	0.287	0.324	0.281	**n/c**				
11	0.286	0.259	0.258	0.258	0.246	0.235	0.275	0.315	0.259	0.242	**0.005**			
12	0.241	0.245	0.250	0.257	0.256	0.242	0.269	0.334	0.254	0.211	0.208	**0.014**		
13	0.271	0.238	0.238	0.240	0.242	0.225	0.284	0.320	0.265	0.240	0.264	0.226	**0.006**	
14	0.272	0.235	0.236	0.251	0.231	0.230	0.254	0.293	0.264	0.206	0.245	0.173	0.214	**0.004**

Bolded values are intraclade P-distance numbers.

### 3.2 28S Phylogenetic analyses

**Fig. 1:**
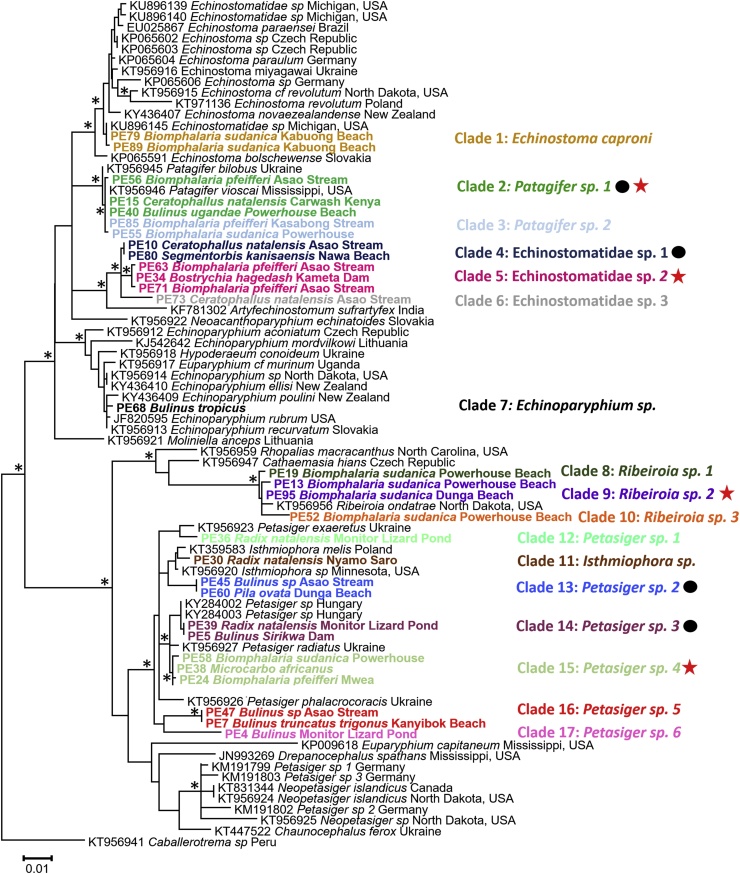
Phylogenetic relationships of echinostomes from this and study (bolded) and from GenBank (with accession numbers) based on 1143 bp of the 28S gene inferred from ML and BI analyses. Nodes with a (*) indicate nodes that were supported (>90%) by bootstrap values and posterior probabilities. Specimens are named based on sample name, the host and locality it was collected from, and color-coded based on clade designation from *nad*1 p-distance values of less than 1.5%. A black circle indicates clades where more than one genus of snails was found to be infected and a red star indicates clades where sequences from two different life-cycle stages matched.

**Fig. 2:**
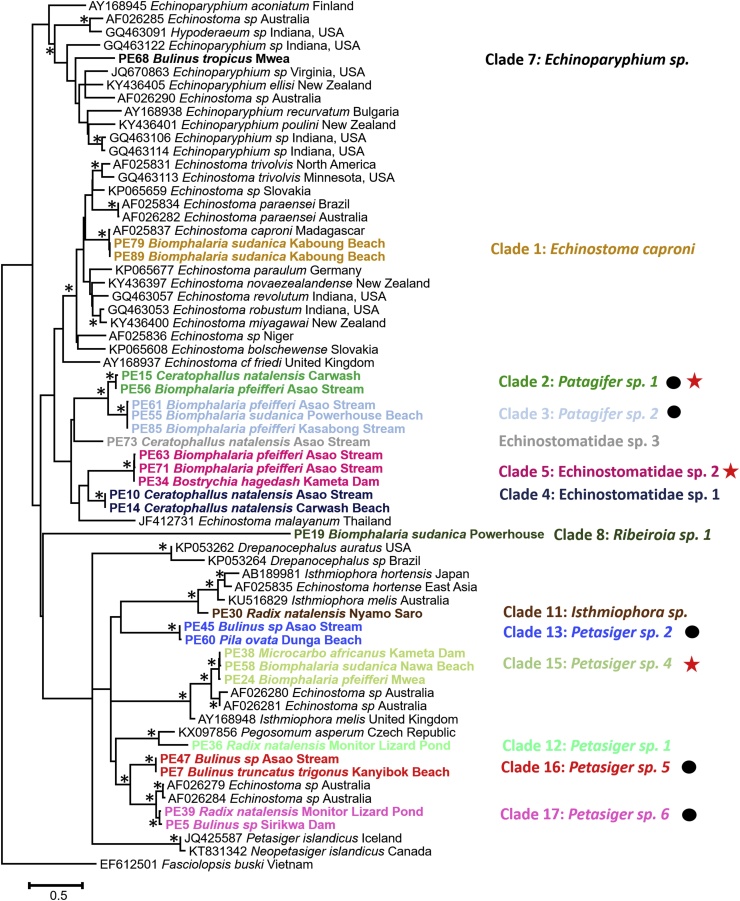
Phylogenetic relationships of echinostomes from this and study (bolded) and from GenBank (with accession numbers) based on 463 bp of the *nad*1 gene inferred from ML and BI analyses. Nodes with a (*) indicate nodes that were supported by (> 90%) bootstrap values and posterior probabilities. Specimens from this study are named based on sample name, the host and locality it was collected from, and color-coded based on clade designation from *nad*1 *p*-distance values of less than 2%. A black circle indicates clades where more than one genus of snails was found to be infected and a red star indicates clades where sequences from two different life-cycle stages matched.

**Fig. 3:**
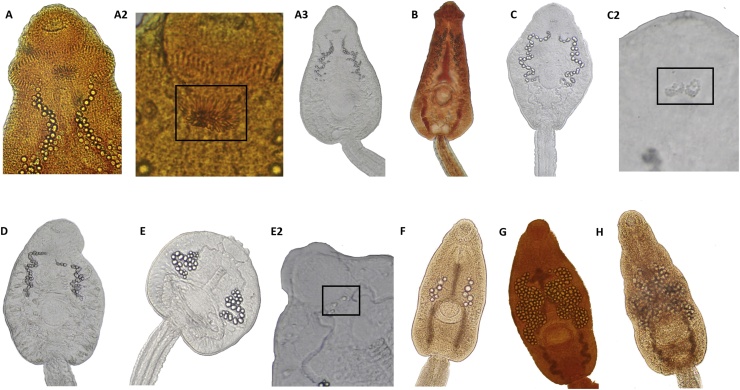
Pictures of echinostomoid cercariae collected from Kenya: Clade 3, *Patagifer* sp. 2 is A1–3. A2 represents the cluster of spines posterior to the oral sucker, (B) clade 1, *Patagifer* sp. 1, (C1–2) clade 5 echinostomatidae sp. 2 and C2 displays the cluster of granules just posterior to the oral sucker, (D) clade 4 echinostomatidae sp.1, (E1–2) clade 14, *Petasiger* sp. 4 and E2 displays the two large granules posterior to the oral sucker, (F) clade 13 *Petasiger* sp. 2, (G) clade 10 *Ribeiroia sp*. 3, and clade 8 *Ribeiroia* sp. 1.

Forty-seven samples from GenBank and 98 specimens from this study were used in phylogenetic analyses to determine how the samples were related. Because some of our resulting clades had multiple representatives, we chose two or three specimens per clade to simplify the presentation of echinostome diversity. Sequences (1243 bp) were obtained for all 98 samples of which 1143 bp were used for Maximum Likelihood (Fig. 1) and BI (not shown) analyses. Analyses were run using the G + I+F model of nucleotide substitution by the Akaike Information Criterion (AIC) jModelTest 2.1 (Darriba et al., 2012). *Caballerotrema sp*. was used as the outgroup because it is the most closely related family to Echinostomatidae that has GenBank records. (Tkach et al., 2016). ML and BI topologies were identical and overall the BI analysis had higher nodal support than the ML analysis. These analyses revealed 17 clades, the names for which are shown in Fig. 1. Clades were color coded (Fig. 2, Fig. 3) based on intraclade *nad*1 *p-distance* value of less than 1.5% (see below).

### 3.3 nad1 Phylogenetic analyses

Forty-one samples from GenBank and the same specimens from this study were used to generate the 28S tree in this study were used in the analysis. Four of the *Ribeiroia* samples did not amplify or the quality of the sequences was poor. Therefore, 94 samples were used in the original analyses and to determine *p-distance* values. *Fasciolopsis buski* (EF612501) was used as the outgroup instead of *Caballerotrema* sp. because *nad*1 sequences for *Caballerotrema* sp. are not represented in GenBank (Tkach et al., 2016). ML and BI analyses were run using the GTR + I+G model of nucleotide substitution by the Alkaike Infromation Criterion (AIC) jModelTest 2.1 (Darriba et al., 2012). The ML and BI topologies were identical and overall the BI tree had higher nodal support than the ML tree. *Nad*1 sequences revealed two additional clades that were not found from the 28S analysis (see below under *Patagifer*).

### 3.4 Clade 1 (Echinostoma caproni)

Two of our specimens (PE79 and PE89) were representatives of *Echinostoma caproni* (*p*-distance value 0.005) based on GenBank accession number, AF025829 from Madagascar (Morgan and Blair, 1998).

### 3.5 Clades 2–3 (Patagifer)

Species of *Patagifer* were known to use ibises as definitive hosts and snails as both the first and second intermediate hosts (Faltynkova et al., 2008). Many of our samples (43) grouped into clades 2 and 3, including 2 samples from Uganda. Thirty-one specimens grouped into clade 2 *(Patagifer* sp. 1) and 12 specimens grouped into clade 3 (*Patagifer* sp. 2). There was a 0.077 (7.7%) *p-*distance value between these two clades. We completed the life cycle of worms from clade 2. We acquired eggs from fecal samples from a sacred ibis (*Threskiornis aethiopicus*), hatched the eggs and experimentally exposed *Biomphalaria sudanica* to the miracidia. We then used cercariae from successful experimental infections to expose *B. sudanica* to obtain metacercariae. We sequenced representatives of each life cycle stage for clade 2 and found them to be identical or to differ by less than 1.0% from one another. Clade 2 cercariae had tail fins and 58–62 collar spines. The larvae also possessed a structure we termed the spine pocket containing approximately 20 spines that was located mid-ventrally just posterior to the oral sucker. Other descriptions called this unit a “brush of needles” (Appleton et al., 1983) or a “rosette of spines” (Ostrowski-de Núñez et al., 1997). These cercariae were also noteworthy for possessing diverticuli (greater than 16/side) along the length of their major excretory canals and for possessing numerous calcareous corpuscles (90–100 granules/side) in each major excretory canal (Fig. 3B). Clade 2 closely grouped with a 28S GenBank sample of an adult *Patagifer vioscai* (KT956946) worm which had 53 collar spines (Faltynkova et al., 2008). Acquisition of *nad*1 sequences for *P. vioscai* from the American white ibis (*Eudocimus albus*), which is endemic to the Americas, would help clarify the relationship to our clade 2 specimens. We also note of interest that our cercariae in clade 2 resembled cercariae from two South American species of *Biomphalaria*: 1) cercariae of *B. tenagophila* from the Uruguay River that transmitted an echinostome cercaria with 58 spines and 16 excretory diverticuli/side (Martorelli et al., 2013), and 2) cercariae from *Biomphalaria straminea* from Argentina have been reported with 53–54 collar spines, a spine pocket, diverticuli and tail fins (Fernandez et al., 2014). Samples from Kazinga Channel in Uganda also grouped into this clade and clade 3.

Clade 3 cercariae have tail fins, 54 collar spines, a spine pocket posterior to the oral sucker containing a cluster of 25 spines (Fig. 3 A2), fewer diverticuli (less than 16/side) along each major excretory canal, and less than 60 calcareous corpuscles within each excretory canal. Appleton et al. (1983) established the life cycle of *Echinoparyphium montgomeriana* from South Africa. He found this species to be transmitted by *Bulinus africanus* and reported it to have 48–54 collar spines and a brush of spines posterior to the oral sucker and was placed in the genus *Echinoparyphium*, which does not correspond to that genus as defined recently by Tkach et al. (2016). Ostrowski de Nunez et al. (1997) described a similar cercaria (that included a spine pocket) transmitted by *Biomphalaria orbignyi* from Argentina with 50 collar spines and less than 16 diverticuli/side associated with each main excretory canal. Lie and Umathevy (1966) described cercariae of *Echinostoma hystricosum* from the lymnaeid snail, *Radix* (*Lymnaea*) *rubiginosa* as having 60 collar spines and a spine pocket as well, but excretory diverticuli were not present.

### 3.6 Clades 4–6 (Echinostomatidae sp. 1–3)

Clades 4–6 did not group closely with any other specimens in GenBank, in either the 28S or the *nad*1 trees. Clades 4 and 5 (Echinostomatidae sp. 1–2) did not have prominent tail fins and have 33 collar spines. Four specimens formed clade 4 and were transmitted by both *Ceratophallus natalensis* and *Segmentorbis kanisaensis*. The cercariae in clade 4 have a cluster of approximately 20 granules just posterior to the oral sucker and approximately forty calcareous corpuscles within each main excretory canal (Fig. 3D). Ten specimens formed clade 5 with only *B. pfeifferi* from a single locality to be shedding this cercaria (Fig. 3C). We also collected an adult worm from a hadada ibis (*Bostrychia hagedash*) that matched the cercariae samples in sequence.

Clade 6 was represented by a single sample of cercariae (PE73) from *Ceratophallus natalensis*, designated Echinostomatidae sp. 3. These cercariae had approximately 18 collar spines on each side and a cluster of about 30 small granules posterior to the oral sucker. Tail fins were not prominent, and many small lipid drops were evident in the body. These cercariae also had approximately 60 small excretory granules in each main canal of the excretory system.

### 3.7 Clade 7 (Echinoparyphium)

A single specimen of a cercaria from *Bulinus tropicus* (PE68) comprised clade 7. The specimen was preserved in ethanol and not maintained in adequate shape to determine the number of collar spines or other morphological features; however, it grouped within sampled identified as *Echinoparyphium* from GenBank samples. There were multiple species descriptions in the literature of *Echinoparyphium* from *Bulinus* from Africa; however, some of the descriptions matched more closely species in *Patagifer* than in *Echinoparyphium* (Appleton et al., 1983). Two species, *E. elegans* and *E. ralphaudyi* were known to be transmitted by *Bulinus* from Africa but there are no samples represented in GenBank and no adult specimens are available for genetic study. It is possible that our specimen was one of these two previously described bulinid-transmitted species based on geography and host-use, but molecular sequences of the two species would be required and finding adults at our study sites to validate this hypothesis.

### 3.8 Clades 8–10 (Ribeiroia)

Five samples from our dataset grouped into three clades of *Ribeiroia* flukes that typically use birds as definitive hosts, planorbids as first intermediate hosts, and amphibians as second intermediate hosts, where they have been reported to cause limb deformities in amphibians (Johnson et al., 2004). The cercariae from *B. sudani*ca in clade 9 (*Ribeiroia* sp. 2) resembled Fain’s (1953) description of *Cercaria lileta* from *Biomphalaria stanleyi*, notable for its possession of a distinctive rose-colored organ placed just posterior to the oral sucker. Based on ITS2 sequences (tree not shown), our cercariae in clade 9 also grouped with sequences derived from cercariae from *B. sudanica* (GenBank AY761143) that also resembled *Cercaria lileta* and possessed the rose-colored organ (Wilson et al., 2005). Our clade 9 samples were from *B. pfeifferi* and *B. sudanica* from central and west Kenya, which resemble earlier descriptions of cercariae from *B. sudanica* (Fain, 1953) and *R. congolensis* which was transmitted by the goliath heron (*Ardea goliath*) from the Democratic Republic of the Congo (Dollfus, 1950; Wilson et al., 2005). In addition, we collected metacercariae (no other larval stages were present) from *B. sudanica* that grouped within clade 9 and that was unexpected because species of *Ribeiroia* are not known for using snails as second intermediate hosts (Johnson et al., 2004).

Cercariae representing clades 8 and 10 developed in *B. sudanica*. Clade 8 (*Ribeiroia* sp. 1, Fig. 3H) was from a single snail (PE19) collected 15 years ago in west Kenya. It had fewer granules in the excretory system than did cercariae of clade 10 (*Ribeiroia* sp. 3). Clade 10 was also represented by a single snail (PE52) of cercariae. These cercariae had a small pharynx and over 120 large, densely packed calcareous corpuscles in each main excretory canal, with some of the corpuscles appearing to be composed of two partially fused corpuscles. These cercariae also had a peculiar organ just posterior to the pharynx. However, this organ lacked the distinctive rose color observed in the cercariae that grouped in clade 9 (Fig. 3G).

### 3.9 Clade 11 (Isthmiophora)

One sample, (PE30) of cercariae from *Radix natalensis* grouped with GenBank records for the genus *Isthmiophora*, which infected small mammals, use molluscs, including lymnaeids, as first intermediate hosts and fish or amphibians as second intermediate hosts (Kostadinova and Gibson, 2002). To our knowledge, this was the first genetic evidence of the genus in Africa

### 3.10 Clades 12–17 (Petasiger)

We found six different clades that likely belonged to the genus *Petasiger.* Members of this genus are known for using snails as first intermediate hosts, fish or tadpoles as second intermediate hosts and birds (mainly cormorants) as definitive hosts (Faltynkova et al., 2008). Cercariae representing all six of the clades we identified had 27 collar spines, which was considered a trait of the genus (Faltynkova et al., 2008). Cercariae representing these clades had two conspicuous refractile granules situated immediately posterior to the oral sucker, an inflated gut and no tail fins. None of these clades matched any GenBank records.

Clade 12 (*Petasiger* sp. 1) was represented by one cercaria (PE36), from *R. natalensis* occurring in central Kenya. It had been preserved for many years in ethanol and it was difficult to make out many of its morphological features for comparison to other specimens in this study or from other published works.

Eight specimens from this study were cercariae from *Bulinus* that grouped into clade 13 (*Petasiger* sp. 2). These cercariae had 7–10 calcareous corpuscles per main excretory canal, a small oral sucker and two refractile granules posterior to the oral sucker (Fig. 3F).

Two specimens, PE39 and PE5 from *R. natalensis* and *Bulinus* sp., respectively formed clade 14 (*Petasiger* sp. 3). Both specimens were collected from central Kenya. The *nad*1 *p*-distance value between these two specimens was 0.014, suggesting that these two specimens were the same species. The cercaria from *R. natalensis* resembled that of an echinostome cercariae described from South Africa also transmitted by *R. (Lymnaea) natalensis* (Moema et al., 2008). Cercariae from both snail hosts had two large granules just posterior to the oral sucker.

The cercariae comprising clade 15 *(Petasiger* sp. 4) that were recovered from *B. pfeifferi* and *B. sudanica* also had two granules just posterior to the oral sucker and 17 calcareous corpuscles in each main excretory canal. Sequences from these cercariae also matched those from an adult worm (PE38) recovered from a reed cormorant (*Microcarbo africanus*). Morphologically this specimen is similar to the *Petasiger* described in Fernandez et al., 2016.

Clade 16 (*Petasiger* sp. 5) likely corresponded to what was described as *Petasiger variospinosus* (King and Van As, 2000) and *Cercaria decora* (Fain, 1953). Cercariae in this clade were both recovered from *Bulinus* sp. Such cercariae had 27 collar spines, two large granules posterior to the oral sucker, and 19–20 calcareous corpuscles in each main excretory canal. The life cycle was completed by experimentally exposing laboratory raised reed cormorants (*Microcarbo africanus*) to metacercariae from *Xenopus* that had been experimentally exposed to cercariae from *B. tropicus* (King and Van As, 2000).

Only one cercaria (PE4) obtained from *Bulinus* sp. comprised clade 17 (*Petasiger* sp. 6). This specimen was from a preserved specimen and it was difficult to make out distinct morphological features.

## 4 Discussion

Analysis of 98 East African echinostome specimens, mostly of cercariae, using 28S and *nad*1 molecular markers revealed 17 clades from 5 genera of freshwater gastropods collected from 19 localities. The boundaries we used to delineate the 17 clades were intraspecific *p*-distance values less 1.5% (*nad*1 gene) and interspecific differences greater than 5% (Vilas et al., 2005). For instance, using *p*-distance values from the *nad*1 gene we could distinguish two distinct species of *Patagifer* (7.7% difference), whereas this distinction was not apparent in our 28S tree or distance matrix.

To help reduce discrepancies between our collected specimens and those in GenBank we used ML and BI analyses to determine how our specimens grouped relative to each other and to echinostomes represented in GenBank and then putatively assigned them a name based on where they grouped. From our analyses, three of the 17 clades (4–6) did not group with any GenBank records. Specimens from clades 4 and 5 possessed 33 collar spines and those from clade 6 had 36 collar spines. There are few previous descriptions of echinostomes with 33 collar spines (Dietz, 1909; Lumsden and Hugg, 1965; Premvati, 1968; Kanev et al., 2009), some of which placed 33-spined echinostomes in either *Echinostoma* or *Petasiger*. However, species of *Echinostoma* have 37 spines (Huffman and Fried, 1990) and *Petasiger* has 27 collar spines (Faltynkova et al., 2008), but our 33-spined samples did not group with either genus (Tkach et al., 2016). Therefore, we did not putatively designate a genus for these clades.

From the addition of our specimens from our survey work in East Africa, we confirmed that *E. caproni* (37-collar-spined group) has a broad distribution throughout Africa (Morgan and Blair, 1998). It is of interest that this species was found because many studies have been done on the immunobiology of *Biomphalaria* and *E. caproni* and others that have shown *E. caproni* rediae move toward intramolluscan stages of other trematodes (Reddy and Fried, 1996). Also, *E. caproni* was dominant against *S. mansoni* in co-infections in *B. glabrata*, and *E. caproni* had enhanced virulence when *B. glabrata* were exposed to both parasites (Sandland et al., 2007). Even though these studies used *B. glabrata* (Neotropical snail), this species is from Africa and uses African *Biomphalaria* as intermediate hosts in nature.

One surprising and previously unappreciated aspect of echinostome biology that emerged from examining a broad spectrum of cercariae was the presence of a variety of peculiar structures lying posterior to the oral sucker. Clades 2 and 3 have a distinctive concentration of spines that appear mid-ventrally, a short distance posterior to the posterior margin of the oral sucker in what we have termed a spine pocket. The 20–30 spines contained in the pocket are similar in size and appearance to the collar spines and are arranged with their bases overlapping centrally and with their sharp distal tips fanning outward and anteriorly. They appear refractile as do the associated collar spines, but the number of collar spines (54–62) for both clades is much greater. A role for the spines in the spine pocket as holdfast structures does not seem likely. Appleton et al. (1983) found the spine pocket of cercariae from *Bulinus africanus* to be lost once the cercariae encyst as metacercariae. Perhaps these spines are somehow moved to a position on the collar to replace spines lost during subsequent encystment as metacercariae or when excysted worms develop into adults in their definitive hosts. One possibility is that the spines in the spine pocket function as a light-harvesting organ to facilitate orientation to light by cercariae once they leave their snail host. As discussed further below, cercariae with spine pockets have also been recovered from South American echinostomes.

Four more peculiar structures were found just posterior to the oral sucker. The second type of peculiar refractile structure was found in clades 4 and 5. The enclosed structure lying just posterior to the oral sucker contains a cluster of granules (20–24), some of which are fused and this feature is similar to other cercariae descriptions by Fain (1953), Lie (1963) and Fernandez et al. (2014). A third type of refractile structure is exhibited by clades 13–16, also which have an enclosed structure located just posterior to the oral sucker. But in the case of clades 13–16, the structure contains only two larger granules, similar to what was described by (Fain, 1953; King and Van as, 1996; King and Van As, 2000, and Moema et al., 2008). A fourth type is found in clade 9, a species of *Ribeiroia* with its cercaria corresponding to *C. lileta* of Fain (1953). Fain (1953) observed a distinctive oval-shaped rose-colored organ just posterior to the oral sucker, the presence of which was confirmed by Wilson et al. (2005) and in the present study. A fifth type, represented by Clade 10, also possessed an identifiable oval structure lying in a comparable position to that seen for *C. lileta,* but it lacked any distinctive coloration. Similar structures have not been described from the many putative species of echinostome cercariae described from North America or Eurasia; however, there are striking morphological similarities between cercariae transmitted by *Biomphalaria* from Africa and South America (Ostrowski-de Núñez et al., 1997; Martorelli et al., 2013; Fernandez et al., 2014) that suggests a historical connection in the southern hemisphere.

Several phylogenetic studies of the genus *Biomphalaria* have indicated that it originated in the Neotropics and later colonized Africa (DeJong et al., 2001). The presence of *Biomphalaria* in South America probably dates to 55–65 million years ago (MYA), whereas its appearance in Africa is relatively recent, <1-5 MYA (Woodruff and Mulvey, 1997; Campbell et al., 2000; DeJong et al., 2001). Given that many echinostome species are hosted by aquatic birds, they may have provided a conduit for dispersal of Neotropical echinostomes to Africa and vice versa (Woodruff and Mulvey, 1997). This idea is supported by the fact that similar cercariae from opposite sides of the Atlantic use related, but distinct species of avian definitive hosts. For example, members of clade 2 from *Biomphalaria* in Africa are known to use sacred ibises as definitive hosts. Their cercariae are remarkably similar to, though distinct from echinostome cercariae from *Biomphalaria straminea* in South America (Ostrowski-de Núñez et al., 1997). There are very few GenBank records of South American echinostomes and further comparisons of sequence data among morphologically similar cercariae between the two continents will help to unravel patterns of intercontinental dispersal or to provide insight if they were part of Gondwanaland.

Exploring the relationships among trematode species that use more than one species and/or genus of intermediate host, for example, is key to understanding the how parasite evolve and persist over time and space. Additionally, such studies can be extended to understand how to manage co-occurring disease-causing parasites, such as *Schistosoma mansoni*. Another interesting aspect among the relationships of the echinostomes and their hosts is the involvement of other planorbid genera and species from both Africa and South America as additional first intermediate hosts. Some of our species of echinostomes recovered from African *Biomphalaria* were sometimes also recovered in another important schistosome-transmitting planorbid genus, *Bulinus*. Using molecular markers, we confirmed that four clades (2, 4, 13, and 14) use more than one genus of snail (and sometimes multiple families of snails) as first intermediate hosts. For example, clade 2 was composed of cercariae samples from *Ceratophallus*, *Bulinus*, and *Biomphalaria*. This finding is in line with another study that also confirmed some echinostomes to have broad first (and second) intermediate host specificity (using multiple genera and families of snails) (Detwiler et al., 2010). In both cases, this diversity of interspecific relationships was not revealed without the use of comparative molecular phylogenetic.

In many cases, it is difficult to complete parasite life cycles because collecting all necessary hosts in a life cycle and experimentally exposing those hosts is most often logistically difficult in most areas. However, using molecular markers we were able to connect at least two hosts (2/3) in the life cycles for four clades of echinostomes. We sequenced life cycle stages (cercariae, metacercariae, or adults) and compared them to one another and if two life cycle stages fell into the same clade in the *nad*1 tree (less than 1.5% pairwise difference) we considered them to be conspecifics. For example, in clade 5, we collected an adult worm from a hadada ibis which fell into the same clade as cercariae from *B. pfeifferi*. Clade 9 was composed of cercariae from *B. sudanica* and *B. pfeifferi* which grouped with metacercariae from *B. sudanica*. We collected an adult from a reed cormorant which grouped with cercariae from *B. sudanica* and *B. pfeifferi* from clade 15. While we do not have complete life cycles for all of the species, we have accumulated life cycle data on the naturally cycling hosts, rather than lab hosts, and we also know that some species can actually use more than one species or family of snail intermediate host.

With respect to transmission of human schistosomiasis, 15 of the 17 clades we found were transmitted by planorbids, suggesting that planorbids are being heavily exploited by these echinostomes even though we collected other snail families including Physidae, Viviparidae, Thiaridae and Bithyniidae for which we did not find any infected with echinostomes. Of the 17 clades, 13 use the same (first) intermediate hosts as human schistosomes (*Biomphalaria* and *Bulinus*). Seven clades are transmitted by *Biomphalaria* and 6 of the clades are transmitted by *Bulinus*. Approximately 44% of the specimens we collected fell into clades 2 and 3 and these clades were transmitted by *B. pfeifferi* and *B. sudanica.* Even though many clades were found to be transmitted by planorbids, we also found 3 of the clades to be transmitted by *Radix natalensis* which is an intermediate host for *Fasciola gigantica* and *F. hepatica*, which causes fascioliasis (Correa et al., 2010). Further investigations should be done on their interactions within *R. natalensis*.

The presence of echinostomes in these snails creates opportunities for competition between other trematode species. Although it is well known that a single snail species can be utilized by multiple different species of digeneans, double infections are rare in nature, and some digenean species interfere with one another’s development within the same intermediate host (Lim and Heyneman, 1972). Dominance hierarchies among digenean species have been documented and certain species of echinostomes have been shown to be dominant among other trematode species (Kuris, 1990; Hechinger et al., 2011). Since 13 of the 17 clades of echinostomes use the same intermediate hosts (first) as human schistosomes, this creates problems for schistosomes to establish in snails because echinostomes (redia) have been shown to be strong competitors against human schistosomes (Lim and Heyneman, 1972; Banes et al., 1974; Rashed, 2002). Because certain echinostome species can be dominant, particularly against human schistosomes, it has been suggested that other larval digeneans can be integrated into schistosome control programs (Bayer, 1954; Lim and Heyneman, 1972; Banes et al., 1974; Pointier and Jourdane, 2000). The use of indigenous echinostome species for control of human schistosomes deserves further consideration, and supplemental studies are needed to ascertain how these African species may affect schistosome abundance. This study provides the first survey list of putative candidates and their relationships to snails to pursue in the control of *S. mansoni* as well as broadening our understanding of parasite community dynamics.

## Financial support

Technical assistance at the University of New Mexico Molecular Biology Facility was supported by the National Institute of General Medical Sciences of the National Institutes of Health under Award Number P30GM110907. We gratefully acknowledge the following agencies for their financial support: The National Institute of Health (NIH) grant R37AI101438, the Fogarty International Center and National Institute of Mental Health, NIH award number D43 TW010543, and the Bill and Melinda Gates Foundation, Seattle, WA (OPP1098449) for the Grand Challenges Explorations Initiative grant. The content for this paper is solely the responsibility of the authors and does not necessarily represent the official views of the National Institutes of Health. This paper was published with the approval of the Director of KEMRI.
